# Active management of the third stage of labour without controlled cord traction: a randomized non-inferiority controlled trial

**DOI:** 10.1186/1742-4755-6-2

**Published:** 2009-01-21

**Authors:** A Metin Gülmezoglu, Mariana Widmer, Mario Merialdi, Zahida Qureshi, Gilda Piaggio, Diana Elbourne, Hany Abdel-Aleem, Guillermo Carroli, G Justus Hofmeyr, Pisake Lumbiganon, Richard Derman, Pius Okong, Shivaprasad Goudar, Mario Festin, Fernando Althabe, Deborah Armbruster

**Affiliations:** 1 UNDP/UNFPA/WHO/World Bank Special Programme of Research, Development and Research Training in Human Reproduction, Department of Reproductive Health and Research, World Health Organization, Geneva, Switzerland; 2 Department of Obstetrics and Gynaecology, Kenyatta National Hospital, University of Nairobi, Nairobi, Kenya; 3 London School of Hygiene and Tropical Medicine, London University, London, UK; 4 Department Obstetrics and Gynaecology, Assiut University Hospital, Assiut, Egypt; 5 Centro Rosarino de Estudios Perinatales, Rosario, Argentina; 6 Effective Care Research Unit, University of Witwatersrand, University of Fort Hare, East London, South Africa; 7 Department of Obstetrics and Gynaecology, Khon Kaen University, Khon Kaen, Thailand; 8 University of Missouri, Kansas City, USA; 9 Department Obstetrics and Gynaecology, San Raphael of St. Francis Hospital Nsambya, Kampala, Uganda; 10 Department of Medical Education, K L E Society's J N Medical College, Belgaum, India; 11 Philippine General Hospital, Manila, Philippines; 12 Department of Mother and Child's Health Research, Buenos Aires, Argentina; 13 Program for Appropriate Technology in Health (PATH), Washington, USA

## Abstract

**Background:**

The third stage of labour refers to the period between birth of the baby and complete expulsion of the placenta. Some degree of blood loss occurs after the birth of the baby due to separation of the placenta. This period is a risky period because uterus may not contract well after birth and heavy blood loss can endanger the life of the mother. Active management of the third stage of labour (AMTSL) reduces the occurrence of severe postpartum haemorrhage by approximately 60–70%. Active management consists of several interventions packaged together and the relative contribution of each of the components is unknown. Controlled cord traction is one of those components that require training in manual skill for it to be performed appropriately. If it is possible to dispense with controlled cord traction without losing efficacy it would have major implications for effective management of the third stage of labour at peripheral levels of health care.

**Objective:**

The primary objective is to determine whether the simplified package of oxytocin 10 IU IM/IV is not less effective than the full AMTSL package.

**Methods:**

A hospital-based, multicentre, individually randomized controlled trial is proposed. The hypothesis tested will be a non-inferiority hypothesis. The aim will be to determine whether the simplified package without CCT, with the advantage of not requiring training to acquire the manual skill to perform this task, is not less effective than the full AMTSL package with regard to reducing blood loss in the third stage of labour.

The simplified package will include uterotonic (oxytocin 10 IU IM) injection after delivery of the baby and cord clamping and cutting at approximately 3 minutes after birth. The full package will include the uterotonic injection (oxytocin 10 IU IM), controlled cord traction following observation of uterine contraction and cord clamping and cutting at approximately 3 minutes after birth. The primary outcome measure is blood loss of 1000 ml or more at one hour and up to two hours for women who continue to bleed after one hour. The secondary outcomes are blood transfusion, the use of additional uterotonics and measure of severe morbidity and maternal death.

We aim to recruit 25,000 women delivering vaginally in health facilities in eight countries within a 12 month recruitment period.

**Management:**

Overall trial management will be from HRP/RHR in Geneva. There will be eight centres located in Argentina, Egypt, India, Kenya, Philippines, South Africa, Thailand and Uganda. There will be an online data entry system managed from HRP/RHR. The trial protocol was developed following a technical consultation with international organizations and leading researchers in the field.

**Expected outcomes:**

The main objective of this trial is to investigate whether a simplified package of third stage management can be recommended without increasing the risk of PPH. By avoiding the need for a manual procedure that requires training, the third stage management can be implemented in a more widespread and cost-effective way around the world even at the most peripheral levels of the health care system. This trial forms part of the programme of work to reduce maternal deaths due to postpartum haemorrhage within the RHR department in collaboration with other research groups and organizations active in the field.

**Trial Registration:**

ACTRN12608000434392

## Background

The third stage of labour refers to the period between birth of the baby and complete expulsion of the placenta. Some degree of blood loss occurs after the birth of the baby due to separation of the placenta. This period is a risky period because uterus may not contract well after birth and heavy blood loss can endanger the life of the mother.

The third stage of labour is managed differently around the world. Over the years two management packages, known as 'active management' and 'expectant management' emerged. The primary aim of active management is to reduce postpartum blood loss as a preventive intervention. In active management (AMTSL) a number of interventions are applied in combination while the expectant management represents a more hands-off style with those interventions used in AMTSL withheld. AMTSL has been defined in various ways and current international definition comprises three components: administration of an uterotonic soon after delivery of the baby; controlled cord traction; and uterine massage after delivery of the placenta [1]. Active management of the third stage of labour (AMTSL) reduces the occurrence of severe postpartum haemorrhage by approximately 60–70% [2].

In previous active versus expectant trials the cord was clamped as soon as possible usually within one minute. However, trials of cord clamping timing have shown beneficial effects on newborn haematological indices leading to the recommendation to clamp the cord at around 3 minutes although the effects on the mother are unknown [3].

The WHO PPH Prevention Guidelines published in 2007 recommended AMTSL defined as the use of oxytocin 10 IU IM/IV after birth, cord clamping at around 3 minutes when the uterus contracts and controlled cord traction. There were no recommendations related to the use of uterine massage in this guideline.

Unfortunately, the relative contribution of the components of AMTSL to the overall reduction in blood loss is not clearly known. Understanding the contribution of the components of AMTSL to the overall effect in reducing the incidence of haemorrhage could have major programmatic significance since some components require training while others require an efficient drug procurement and utilization system [4]. At the same time the probability of interaction between AMTSL components should be kept in mind.

These considerations led to a technical consultation of scientists working in the field of postpartum haemorrhage research in December 2007 to discuss work on a research project to evaluate the effects of different components of AMTSL. Such research is important and timely because some components such as the uterotonic and controlled cord traction may not be feasible in all settings and it is worthwhile evaluating a more simple package with fewer components.

### Systematic reviews of effects of AMTSL components

#### Uterotonic

The uterotonic component of AMTSL seems to be important for the reduction in blood loss after delivery [5]. Two trials compared oxytocin to nothing without other active management components practiced [6,7] and one trial compared oxytocin to nothing with other active management components practiced in both groups [8]. Oxytocin used at 5–10 IU seemed to reduce the risk of severe postpartum haemorrhage although the other components were not well described.

#### Controlled cord traction

Cord traction was introduced into obstetric practice by Brandt (1933) and Andrews (1940) by the so-called Brandt-Andrews maneuver. The aim is to facilitate the delivery of a placenta that is already separated. In 1962 the term *controlled cord traction *(CCT) was introduced which aims to facilitate the separation of the placenta once the uterus contracts [9]. In performing CCT placental separation is not waited and once the uterus contracts the CCT is initiated. The third stage is usually completed in less than 10 minutes when CCT is used.

There is concern by clinicians, based on teachings from their pre-service education that traction on the cord prior to placental separation may lead to maternal complications such as separation of the cord from the placenta and uterine inversion. There is not a large body of direct evidence for or against effects of controlled cord traction in isolation [10].

CCT requires training to acquire this manual skill. Evaluation of CCT component is important because if it does not add any beneficial effects it can be dropped from the AMTSL package with important programmatic implications.

#### Uterine massage

Uterine massage is used following delivery in various forms. Between delivery of the baby and the placenta a birth attendant will often put his/her hand over the uterus to ensure that there is no undiagnosed twin, to assess if the uterus is contracting and may rub the uterus to stimulate contraction. The Cochrane review on active versus passive management does not refer to the use of uterine massage as part of AMTSL whereas the FIGO/ICM statement on AMTSL does include uterine massage as part of AMTSL. Evidence that massage contributes to a reduction in blood loss would provide evidence to support the use of this intervention with simple instructions.

The National Institute for Health and Clinical Excellence of the United Kingdom published comprehensive guidelines on intrapartum management on 26 September 2007  and has made no reference to uterine massage.

A small pilot trial (n = 200) conducted in Assiut, Egypt, used 'sustained uterine massage' started after delivery applied every 10 minutes and continued for 60 minutes. The findings are promising, since women receiving massage had less blood loss > 500 ml and received less additional uterotonics than women not receiving uterine massage [11]. In the two surveys conducted by Prevention of Postpartum Hemorrhage Initiative (POPPHI) massage before placental delivery was practiced in about one third of all deliveries. In the same surveys massage after placental delivery was used in 80–90% of women although it was not possible to observe how long after delivery the massage was continued [12].

Williams Obstetrics states "massage is not employed but the fundus is frequently palpated to make certain that the organ does not become atonic and filled with blood from placental separation". In the United States and some other countries, palpating the uterus and massaging if "soft" for the first few hours after childbirth is considered standard of care [13]. In many countries however, no well-defined massage protocol exists. A systematic review to evaluate the effects of sustained uterine massage from the time of birth of the baby currently contains very little evidence to guide practice [14].

#### Other interventions

One intervention that has recently been included in AMTSL package is delayed cord clamping. There is evidence that delayed cord clamping increases newborn haematological indices in the short term and also has beneficial effects of reduced intraventricular haemorrhage when applied to preterm infants. However, there is almost no evidence on maternal effects especially whether it increases the blood loss.

Other third stage interventions include squeezing the uterus from the fundus to expel the placenta (Credé maneuver) [15], placental cord drainage, umbilical vein injection and variations in timing, dose, and route of above discussed components.

### Rationale for selecting the experimental intervention

An important aspect of AMTSL components is that one cannot assume that each component has no interaction with others. Such interactions could occur for single, double or triple components. The implication is that even if an intervention may not be (as) effective when individually applied it may have effect together with another component. For example, oxytocin and massage could act synergistically.

The relative importance of all of these uncertainties and the feasibility of evaluating the effects of one or more of the components were discussed in detail at the technical consultation referred above in December 2007. The evaluation of two components namely CCT and uterine massage after placental delivery was given priority. The feasibility of evaluating both interventions in one trial was discussed in detail. Different epidemiological designs such as three-arm and 2 × 2 factorial design were considered. Evaluating more than one component in a single trial posed particular difficulties. Such difficulties originated from the nature of the intervention(s) (manual procedures being difficult to mask) and the low incidence of the primary outcome measure. The implications of these two factors were resorting to cluster or cross-over cluster designs with added complexity and large sample sizes. At the end of the consultative process evaluation of a package that does not include controlled cord traction was regarded as the highest priority research question among all discussed. The main reason for this conclusion is that currently, the global scale up of AMTSL is limited to those settings where skilled birth attendants are available largely because controlled cord traction (CCT) requires clinical skills training. Considerable training investments are needed to enable health providers to do CCT correctly and safely.

The presence of a skilled birth attendant is essential in every childbirth. Birth attendants are required for not only basic care during the first and second stage of labour but also in the third stage when haemorrhage risk is greatest and also for the immediate care of the newborn. We aim to identify the relative effect of one component that requires manual skill training. In the most peripheral points of the health system the availability of health personnel with skills to implement the full AMTSL package may be more difficult to ensure in some settings. Therefore, evaluation of whether a simplified package without CCT is non-inferior to the full AMTSL package has both clinical and public health importance. The simplified package is also less intrusive for the mother.

If it were possible to demonstrate the non-inferiority of a simplified form of AMTSL, dispensing with CCT, it would greatly reduce programme costs associated with training, and enable the intervention to be used by lower level providers, especially in out-of-hospital settings.

We therefore put forward the hypothesis that a simplified package comprising oxytocin 10 IU IM/IV (with or without uterine massage) is as effective as the full AMTSL package.

### Objective

The primary objective is to determine whether the simplified package of oxytocin 10 IU IM/IV, without CCT, is not less effective than the full AMTSL package with regard to reducing blood loss ≥ 1000 ml in the third stage of labour. The hypothesis will be that of non-inferiority within a margin of 0.45 to 0.50%. If this hypothesis is demonstrated, the simplified package without CCT could be adopted, with the advantage of not requiring training to acquire the manual skill to perform this task.

### Study design

This will be a hospital-based, multicentre, individually randomized non-inferiority controlled trial.

Each woman will be randomized to receive either the full (AMTSL) package or the simplified package. The blinding of the intervention will not be possible because the comparison includes a manual procedure.

#### Interventions

In order to overcome the variations in the actual practice of AMTSL a working definition was discussed and agreed upon during the technical consultation.

#### Experimental arm: Simplified package

The simplified package will include;

##### • Uterotonic

Oxytocin 10 IU IM. Oxytocin will be administered as soon as possible after birth preferably within one minute. If a woman has an IV line oxytocin can be administered through the IV line by slow injection.

##### • Cord clamping

Cord will be clamped following observation of a uterine contraction either manually or visually. For practical purposes this is estimated to be around 1–3 minutes. It is recommended to clamp the cord close to the perineum.

##### • Placental delivery

Cord traction is omitted. Placenta should be delivered passively by aid of gravity and maternal effort. The caregiver should observe the placental separation and as the placenta delivers it should be held in two hands and gently turned so that the membranes do not tear off.

##### • Uterine massage

No uterine massage is recommended before placental delivery. After placental delivery the uterus will be rubbed and any clots expressed. For a period of two hours the uterus will be massaged gently until it contracts and this procedure will be repeated every 15 minutes. It is acknowledged that not all centres will be able to implement this component. Each centre will decide before the trial begins whether the uterine massage component will be implemented or not.

#### Standard intervention arm: Full AMTSL package

The full package will include:

##### • Uterotonic

Oxytocin 10 IU IM. Oxytocin will be administered as soon as possible after birth preferably within one minute. If a woman has an IV line oxytocin can be administered through the IV line by slow injection.

##### • Cord clamping

Cord will be clamped following observation of a uterine contraction either manually or visually. For practical purposes this is estimated to be around 1–3 minutes. It is recommended to clamp the cord close to the perineum.

##### • Placental delivery

Placenta will be delivered by controlled cord traction immediately after cord clamping and cutting. The cord will be gently pulled while applying counter traction to the uterus with the other hand. As the placenta delivers it should be held in two hands and gently turned so that the membranes do not tear off.

##### • Uterine massage

No uterine massage is recommended before placental delivery. After placental delivery the uterus will be rubbed and any clots expressed. For a period of two hours the uterus will be massaged gently until it contracts and this procedure will be repeated every 15 minutes. It is acknowledged that not all centres will be able to implement this component. Each centre will decide before the trial begins whether the uterine massage component will be implemented or not.

The two intervention packages will differ only in placental delivery technique. If the umbilical cord has been clamped early because of newborn indication cord traction should be applied only after the uterus has contracted as above.

#### Outcome measures

The primary outcome measure is blood loss of 1000 ml or more at one hour and up to two hours for women who continue to bleed after one hour. Blood collection will be made using the calibrated drape BRASSS-V^® ^and measured from delivery to one hour following delivery or up to two hours if bleeding continues [16].

Secondary outcomes are;

• blood transfusion,

• the use of additional uterotonics to treat PPH

• blood loss 500 ml or more

• postpartum maternal haemoglobin (in centres where feasible)

• maternal death

• manual removal of placenta,

• additional surgical procedures (e.g. hysterectomy, ligation of vessels)

• composite outcome of maternal death or severe morbidity (admission to intensive care unit, hysterectomy, blood loss of two liters or more, uterine inversion, near miss event as defined in the manual of operations)

• initiation of breastfeeding

• side effects such as nausea and abdominal pain

Once the cord is clamped and cut the drape will be placed under the woman's buttocks and blood loss measurement will begin. The blood loss measurement will continue for one hour regardless of whether the woman is kept in the delivery room or moved elsewhere. If the bleeding continues beyond one hour the blood loss measurement will continue until two hours postpartum.

Side effects will be recorded by the researcher. Any side effect requiring treatment will be regarded as an adverse event and a separate form will be filled.

Serious adverse events will be recorded in special forms and will be returned by the local investigators to the trial coordination unit within 24 hours.

#### Study population

All women expecting to deliver vaginally at the participating hospitals will be potentially eligible. Women will be approached primarily during antenatal care and early labour for consent for participation except under the following circumstances;

1. Advanced first stage of labour (> 6 cm cervical dilatation)

2. Women who are too distressed to give consent regardless of cervical dilatation or phase of labour. Such evaluation will be made by the clinician in charge of the care of the woman.

3. Minors without a guardian

4. Planned caesarean section

5. If the birth is considered an abortion according to local limits

6. Women with twins or higher order multiple gestations

7. Women who are not capable of giving consent due to other health problems such as obstetric emergencies (e.g. eclampsia) or mental disorder.

Women receive AMTSL regardless of whether they are at high or low-risk for haemorrhage. Therefore all women will be eligible for participation according to the exceptions mentioned above.

## Methods

### Generation of allocation sequence

The random allocation sequence will be generated centrally at WHO Headquarters using computer generated random numbers. Randomization will be to two groups and stratified by country. Blocking with randomly varying groups of 6–8 will be used to restrict randomization within the strata.

### Random allocation technique and allocation concealment

Allocation of the random generated sequence will be by consecutively numbered envelopes. Allocation concealment will be achieved by using sealed opaque envelopes. Allocation will take place during second stage when vaginal delivery is imminent. Once the envelope is opened the name of the woman will be entered on the log file with the envelope number and that woman is enrolled in the trial.

### Rationale for the non-inferiority hypothesis and for sample size estimation

The objective of this trial is to show 'non-inferiority' of the simplified package' compared to the full AMTSL package. As such, while superiority would be an added bonus it is not expected and is probably not plausible since the experimental intervention has fewer interventions.

In the conventional superiority trial, the aim is to determine whether one intervention is superior to another, for example, whether uterotonic is superior to nothing. By contrast, in a non-inferiority trial, the aim is to determine whether an alternative intervention with certain advantages is similar to a gold standard. The full AMTSL package represents the gold standard management strategy for reducing blood loss in the third stage of labour. In order to evaluate the effectiveness of a simplified package with fewer components that package has to be compared to the full package to see whether t is "non-inferior" to the gold standard [17].

Because proof of exact equality is impossible, a pre-stated margin of non-inferiority (Δ) for the difference in effectiveness has to be defined. The choice of the non-inferiority margin can be made using clinical assessment, which is to a certain extent arbitrary, and needs consensus among different stakeholders. A technique which has been proposed [17] to establish the non-inferiority margin is to look at the effect of the gold standard, in this case the full AMTSL, compared with placebo in historical (past) trials, in this case the expectant management. A reasonable criterion is to preserve 80% of the benefit of the full AMTSL package (considered as 100%) over expectant management (considered as 0%). Preserving a higher percentage (say 90%) will push the sample size calculations very high while a smaller percentage (say 50%) may not be considered acceptable.

Estimates of blood loss ≥ 1000 ml (severe PPH – sPPH) with active and passive management were taken from the literature. In addition, unpublished data from several ongoing WHO studies where postpartum blood loss is measured were obtained. Based on those data, a 1.5% risk of blood loss of 1000 ml with full active management was considered realistic and appropriate.

The expectant management sPPH rate was more difficult to estimate because the data was only available from earlier published trials and blood loss estimation methods and the actual expectant management method differed between trials. Values between 3.0 to 4.0% are considered realistic and used for sample size estimations.

Table 1 includes different scenarios for the calculation of sample size, obtained by varying the sPPH rate with expectant management (3.0 to 4.0%) and varying the % retained effect (70% to 80%), assuming a fixed 1.5% sPPH rate for the full AMTSL package. These different scenarios result in different choices of the margin of non-inferiority **Δ**. The total sample size required for a non-inferiority test at the 2.5% level using a two-sided 95% confidence interval and a power of 80% is shown, as well as the power obtained for fixed sample sizes of 20,000 and 25,000.

**Table 1:** Sample size scenarios

Blood loss ≥ 1000 ml with full amtsl	Blood loss ≥ 1000 ml with Expectant	Estimated impact of Full AMTSL	Proportion (of full AMTSL effect) retained by the simplified package	Δ (Difference to exclude to claim equivalence)	Total N for 80% Power	Power with total N = 20,000	Power with total N = 25,000
1.5	3.0	1.5	0.7	0.45	22,908	75%	83%

1.5	3.5	2.0	0.75	0.50	18,555	83%	90%

1.5	4.0	2.5	0.8	0.50	18,555	83%	90%

A trial of 22,908 women will have 80% power to show non-inferiority of the simplified package within 0.45% of the full AMTSL package sPPH rate (i.e. less than 1.95% sPPH rate), with a level of significance of 2.5% (Table 1, row 1). The estimates from the centres that have expressed interest in participating in the trial indicate that approximately 20,000–25,000 women can be recruited in a 12 months recruitment period. These centres have collaborated with WHO before and have experience in third stage trials.

### Type of data and data collection procedure

The trial will be conducted in the labour ward of maternity facilities. All women with anticipated vaginal delivery will be eligible. During the trial period information on the total number of births in the facility will be collected to monitor the recruitment efficiency. Ideally, the participating centres should enroll women 24 hours a day. However, such recruitment may not be possible at all centres.

The main data collection will be during the immediate postpartum period but women will be followed up until discharge from the hospital to record any additional interventions and complications that may occur. Once the bleeding stops and the woman is ready for transfer to the postnatal ward the researcher will fill the data collection form. After discharge the data collection forms will be completed and the form data will be entered on to the online system. The online data entry will be either at the hospital or country level depending on local capacity.

### Analysis plan

The main analysis will be on an intention-to-treat principle with comparisons made between the 'simplified' and 'full' AMTSL packages for primary and secondary outcomes. An alternative to the ITT analysis (non-ITT analysis, sometimes called 'per-protocol'), that excludes patients not on their intended treatment and/or those who are not protocol-compliant in any other way could bias the trial in either direction, depending on which patients are thus excluded. In non-inferiority and equivalence trials, performing non-ITT analysis in addition to ITT analysis is desirable as a protection from the increase in type I error risk.

We expect only a small number of protocol deviations because the personnel involved in the study will receive intensive and continuous training and because the trial will be closely monitored by trial investigators and periodically by a Data Monitoring Committee that will look at protocol deviations. Thus, the decrease in power due to exclusions of protocol deviations will be minimal. Table 1 shows that for the proposed sample size, the power is higher than the usually required 80%.

There may be deviations from the trial protocol if the baby's condition requires urgent action. In such situations the cord may be clamped and cut immediately after birth of the head and if the caregiver is involved in the resuscitation the timing of placental delivery may differ (this could be in either direction). We would expect this deviation to be equally distributed in the two groups. A second type of deviation could occur if a woman who has earlier given consent and randomly allocated to one group withdraws her consent after randomization. The withdrawal of consent after random allocation should be extremely rare since the random allocation will be made when vaginal delivery is imminent We have looked at earlier trials where expectant management was used (because expectant management omits controlled cord traction) and did not find any suggestion that there was a compliance problem with omission of CCT. Nevertheless, the sample size calculation has a margin of additional power as mentioned in table 2 to ensure that such protocol deviations do not have a negative impact on the conclusions[17]

**Table 2:** CONSORT recommendations in the protocol – Checklist for non-inferiority and equivalence trials. Items 1 through 12[17]

Paper section and topic	Item	Descriptor	Reported on section
TITLE & ABSTRACT	1	How participants were allocated to interventions (*e.g*., "random allocation", "randomized", or "randomly assigned"), *specifying that the trial is a non-inferiority or equivalence trial*.	Title in cover page. Summary: section 1

*INTRODUCTION *Background	2	Scientific background and explanation of rationale, *including the rationale for using a non-inferiority or equivalence design*.	2 and 3. In particular, see 2.1.5, two last paragraphs for rationale for non-inferiority

*METHODS *Participants	3	Eligibility criteria for participants *(detailing whether participants in the non-inferiority or equivalence trial are similar to those in any trial(s) that established efficacy of the reference treatment) *and the settings and locations where the data were collected.	4.3 and 4.4.3

Interventions	4	Precise details of the interventions intended for each group *detailing whether the reference treatment in the non-inferiority or equivalence trial is identical (or very similar) to that in any trial(s) that established efficacy*, and how and when they were actually administered.	4.1

Objectives	5	Specific objectives and hypotheses, *including the hypothesis concerning non-inferiority or equivalence*.	3

Outcomes	6	Clearly defined primary and secondary outcome measures *detailing whether the outcomes in the non-inferiority or equivalence trial are identical (or very similar) to those in any trial(s) that established efficacy of the reference treatment *and, when applicable, any methods used to enhance the quality of measurements (*e.g*., multiple observations, training of assessors).	4.2

Sample size	7	How sample size was determined *detailing whether it was calculated using a non-inferiority or equivalence criterion and specifying the margin of equivalence with the rationale for its choice*. When applicable, explanation of any interim analyses and stopping rules (*and whether related to a non-inferiority or equivalence hypothesis*).	4.4.3

Randomization – Sequence generation	8	Method used to generate the random allocation sequence, including details of any restrictions (*e.g*., blocking, stratification)	4.4.1

Randomization – Allocation concealment	9	Method used to implement the random allocation sequence (*e.g*., numbered containers or central telephone), clarifying whether the sequence was concealed until interventions were assigned.	4.4.2

Randomization – Implementation	10	Who generated the allocation sequence, who enrolled participants, and who assigned participants to their groups.	4.4.2

Blinding (masking)	11	Whether or not participants, those administering the interventions, and those assessing the outcomes were blinded to group assignment. If done, how the success of blinding was evaluated.	4 (last sentence before 3.1)

Statistical methods	12	Statistical methods used to compare groups for primary outcome(s), *specifying whether a one or two-sided confidence interval approach was used*. Methods for additional analyses, such as subgroup analyses and adjusted analyses.	4.4.3 & 4.5

The actual management of third stage of labour will be recorded in all women to enable the analysis mentioned above.

Comparisons will be expressed as relative risks and risk differences with 95% confidence intervals. The analysis will be stratified by site, and if there is heterogeneity between the sites for any of the results possible causes of heterogeneity will be explored. In the presence of heterogeneity, the results will not be combined in a single summary estimate (or pooled estimate). In the absence of heterogeneity at the 5% level, the results will be combined.

**Figure 1:**
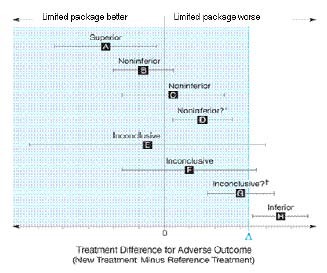
**Scheme for the interpretation of the results of the primary analysis**.

The primary analysis will follow the following scheme for interpretation (Figure 1):[18] If the lower limit of the 95% confidence interval lies to the right of the non-inferiority margin (Δ) the experimental intervention will be declared "inferior" (situation H). Situations A-D indicate non-inferiority with respect to the pre-stated margin Δ. Situations E-G are inconclusive, although E and F are not likely to occur because the trial size guarantees sufficient power. If the results are inconclusive regarding the non-inferiority hypothesis and the whole 95% confidence interval lies to the right of zero (situation G), it will be concluded that the limited package was statistically significantly worse according to a superiority hypothesis.

#### Interim analysis

A Data Monitoring Committee (DMC) with no direct involvement in the trial will be appointed. The role of the DMC will be to deal with any ethical issues that may arise while the trial is in progress, and to scrutinize an interim analysis. An interim analysis will be conducted to be reviewed by the DMC after the completion of the first 5,000 participants. A second interim analysis will be conducted after 10,000 – 15,000 women are recruited. Interim analyses will be masked to trial investigators but not to DMC members. Reporting and handling of adverse events will be in accordance with the Good Clinical Practice guidelines.

#### Stopping the trial

The DMC will be asked to give advice regarding stopping the trial if they have proof beyond doubt of an important advantage or disadvantage for one of the treatment groups, and they consider that the results are likely to affect clinical practice. For the main outcome results of the interim analyses, the DMC will base their recommendation on a superiority hypothesis two-sided stopping rule: if one of the treatments is significantly superior to the other, they will consider to stop the trial. The Haybittle-Peto rule for the α-spending function will be used to determine the level of significance at the two interim analyses and at the final analysis.

#### Stratified analysis

It is likely that some centres will practice uterine massage and some not. A stratified analysis will be conducted of the primary outcome by the use of uterine massage, to assess the interaction of uterine massage by CCT. However, it is likely that such an analysis will be underpowered. The use of uterine massage will also be considered as a potential confounding variable.

## Duration of the project

It is anticipated that the recruitment into the study in the centres can be completed in approximately 12 months. Recruitment will begin around November-December 2008 after trial procedures have been tested and materials have been procured and distributed to the study sites. (Calibrated drape BRASS-V and oxytocin).

## Expected outcomes of the study

The main objective of this trial is to investigate whether a simplified package of third stage management can be recommended without increasing the risk of PPH. By avoiding the need for a manual procedure that requires training, the third stage management can be implemented in a more widespread and cost-effective way around the world even at the most peripheral levels of the health care system.

## Competing interests

The authors declare that they have no competing interests.

## Authors' contributions

All authors contributed to the development of the trial protocol and approved the submitted manuscript. AMG is the guarantor of the manuscript
